# Changes in the Strength and Leaching Characteristics of Steel Slag-Oil Shale Residue-Based Filling Paste in a Complex Erosive Environment

**DOI:** 10.3390/ma16134593

**Published:** 2023-06-25

**Authors:** Fengmei Lian, Chuanyang Du, Dan Meng

**Affiliations:** 1 College of Architecture and Transportation, Liaoning Technical University, Fuxin 123000, China; 2 School of Civil Engineering, Liaoning Technical University, Fuxin 123000, China; 3 School of Built Environment, University of New South Wales, Sydney, NSW 2052, Australia

**Keywords:** cemented paste backfill, consisting of total solid waste, NaCl erosion, dry–wet cycles, oil shale residue, Friedel’s salt

## Abstract

Our research group prepared a new filling paste consisting of steel slag–oil shale residue and no admixtures. It was used as the research object to explore the combined effect of chloride and dry–wet cycling-driven erosion on the long-term stability of a cemented filling paste made of total solid wastes. Macroscopic experiments and microscopic analyses methods were employed. The influence of solutions with different mass fractions of chloride salts and different cycling periods on the uniaxial compressive strength and toxicity of the steel slag–oil shale residue-based filling paste was studied, and the deterioration mechanisms of the steel slag–oil shale residue-based filling paste under combined erosion from chloride and dry–wet cycling were investigated. The test results showed that in the same cycling conditions, the strength of the steel slag-oil shale residue-based filling paste increased first with the increase in the mass fraction of the chloride solution and then decreased with the increase in the mass fraction of the chloride solution after reaching the peak value; the leached concentrations of heavy metal ions decreased with increasing chloride salt mass fraction. With an increase in the number of dry–wet cycles, the compressive strength of the specimens in the chloride salt solution with a mass fraction of 0 (pure water) first increases and then tends to be stable. The strength of samples in 5% and 10% chloride salt solutions increased first and then decreased with an increase in the number of dry–wet cycles. The leached concentrations of heavy metal ions from the samples in all three solutions first decreased and then stabilized. The prehydration products of the steel slag–oil shale residue-based filling paste were C-S-H gels, AFt and Friedel’s salt, and these increased with increasing chloride salt mass fraction and the number of dry–wet cycles. However, the hydration reactions of the samples in the 0% chloride solution nearly stopped in the later stages of cycling, and the samples in 5% and 10% chloride salt solutions developed local cracks due to the accumulation of hydration products. The results showed that the number of dry–wet cycles and the chloride salt mass fraction affected the strength and leaching characteristics of the steel slag–oil shale residue-based filling paste by changing the type and amount of erosion products. The test results provide a scientific basis for the promotion and application of backfilling pastes made from total solid wastes.

## 1. Introduction

With the rapid development of the world’s economy, the scale and intensity of mining have reached an unprecedented level [1]. Large-scale mining has resulted in a sharp increase in the number of goafs and in increasingly serious incidences of surface subsidence. At the same time, the large amounts of solid wastes that are stored on the ground surface not only occupy land but also cause substantial damage to the environment [2]. Mine filling methods have been widely used in recent years because they can be effectively used to fill goaf areas, limit surface collapse and realize the sustainable development of the mining industry [3,4]. Among them, paste filling technology can effectively solve safety problems in the mining process and prevent environmental pollution caused by open-pit solid waste accumulation; therefore, it has emerged as one of the best mine filling methods [5]. Traditional filling methods that use cement as a gelling agent are not in line with the current goals to reduce carbon emissions and yield environmentally friendly products, and they result in high costs. As a result, these filling methods are being replaced with new, green mine filling technologies. Total solid waste filling pastes contain only solid wastes and water, and through the synergistic action of solid waste and water, a cemented filling paste with favorable hydraulic properties is formed. The solid waste is utilized in a way that is efficient and reduces carbon emissions, and the goal of “addressing two risks with one product” can be realized, an approach that has attracted increasing attention [6]. However, after mine filling, the filling paste is eroded by mine water [7]. Cl^−^ is a common and abundant ion in mine water [8]. Due to its strong permeability, Cl^−^ can enter fissures in filling pastes and react with internal hydration products, resulting in deterioration of the filling paste. In addition, groundwater levels fluctuate as a result of rainwater infiltration, and the filling pastes often undergo dry–wet cycling [9]. The combined effect of chloride and wet–dry cycling-driven erosion on the mechanical and chemical stability of solid waste filling pastes is still unclear. Therefore, it is of great practical significance to study the mechanical strength and leaching characteristics of solid waste filling pastes under the combined action of chloride erosion and dry–wet cycling.

Some scholars have done a lot of research on the performance changes of cement-based filling pastes under chloride erosion and wet-dry cycling erosion conditions. Du et al. [10] used cement, fly ash and coal gangue as raw materials to prepare a filling paste and found that Friedel’s salt formation in the test specimen was a key factor affecting strength when the specimen was soaked in a chloride solution. Zhou et al. [11] took cement-based composite materials as the test object, studied the changes in the physical and mechanical properties of specimens under the action of wet–dry cycles and found that long-term wet–dry cycles caused irreversible damage to cement-based composite materials. Wang et al. [12] prepared a filling paste with cement, slag, desulfurized gypsum and other materials and studied the failure trends in the filling paste under the combined action of dry–wet cycles and chloride ions. Filling pastes remain immersed in mine water over long periods of time, and heavy metal ions present in the pastes may migrate to groundwater and cause environmental pollution [13]. Chen et al. [14] explored the variations in the leached ion concentration of a cement-phosphypsum filling paste with temperature and found that hydration products such as AFt and C-S-H gels inside the filling paste had an adsorptive effect on ions, and temperature affected ion leaching by changing the type and quantity of hydration products. Mahedi et al. [15] conducted four kinds of leaching tests to study the leaching characteristics of cement-activated fly ash and slag–treated soil and found that the ion leaching mechanism mainly involved the dissolution and precipitation of oxides and hydroxides. Yang et al. [16] used slag, steel slag and cement as cementing materials combined with fly ash and mine tailings to prepare a filling paste and found that the filling paste immobilized Cd^2+^ significantly under the optimal mixing ratio.

Scholars from all over the world have carried out a large number of studies on the selection of filling pastes, the mechanical properties of filling pastes, and paste erosion driven by chloride and dry–wet cycling. Most studies have employed cement-based materials with admixtures and alkali activators, while few studies have been conducted on cemented filling pastes containing solid wastes without admixtures, particularly with respect to the durability of solid waste filling pastes under the action of chloride erosion, dry–wet cycling and other complex environmental conditions. The mechanical strength and leaching characteristics of solid waste filling pastes in a complex environment may be different from those of traditional filling pastes. In this paper, a cemented backfilling paste consisting of total solid wastes and no admixtures that was developed by our research group was taken as the research object. Through laboratory tests, the compressive strength and leaching characteristics of this cemented paste backfill under the combined action of chloride erosion and wet–dry cycling were studied. In addition, SEM and XRD analyses were combined to explore the variations in the material’s microstructure and phase composition and reveal its deterioration mechanism. This study provides a scientific basis for the promotion and application of this cemented paste backfill technology.

## 2. Materials and Methods

### 2.1. Materials

**Figure 1:**
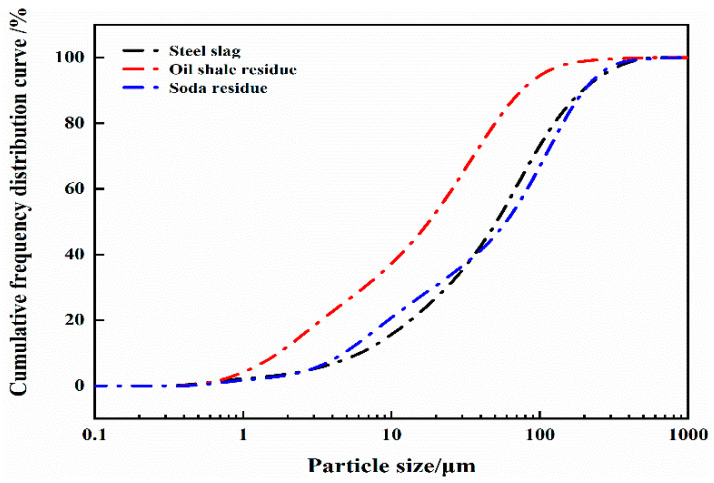
Particle size distribution curve of raw materials.

The steel slag used in this research was taken from the Anshan Iron and Steel Group. Before use, the steel slag was ground, mixed evenly with a vertical planetary ball mill (SM-500, Nanjing, Jiangsu, China), sealed and stored. Oil shale residue was obtained from the west Fushun open pit. Soda residue was obtained from a chemical plant in Dalian and dried in an oven at 60 °C. It was ground into a powder by an ore crusher (FS-200, Zhengzhou, Henan, China) and sealed for storage. The specific surface areas of steel slag, oil shale residue and soda residue are 0.497 m^2^/g, 1.190 m^2^/g and 0.54 m^2^/g, respectively. Figure 1 shows the particle size distribution curves of the three raw materials, Table 1 shows the main mineral compositions and Table 2 shows the quantities of Fe^2+^, Mn^2+^, Cu^2+^ and Zn^2+^ in the raw materials. The nitric acid solution and sodium chloride crystals used in the tests, both produced by Sinopharm Chemical Reagent Co., Ltd. (Shanghai, China), were analytically pure (AR). In the preparation of the filling system, tap water from a municipal distribution network was used as the mixing water, and deionized water was used as the extraction solution in the leaching tests.

### 2.2. Methods

**Figure 2:**
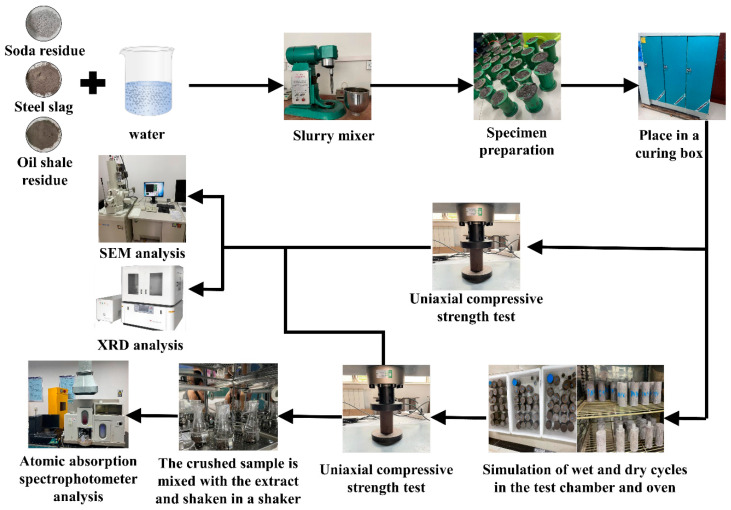
Test flow chart.

The main test contents include preparation and curing of the specimen, uniaxial compressive strength test, combined chloride and wet–dry cycle erosion tests, toxic substance leaching test and microscopic analysis. The test process is shown in Figure 2.

#### 2.2.1. Preparation and Curing

According to previous results from our research group [17], the mix ratio was optimized considering the strength, the degree of collapse and the cost of the filling paste. The optimal ratio of the solid mass fraction was 70.32% and of the water mass fraction was 29.68%, and steel slag, oil shale residue and soda residue were 47.43%, 40.54% and 12.03% of the total solid mass, respectively. The mixtures were added into a mixer according to the optimal ratio and stirred for 2 min. After mixing evenly, the mixed water was admixed into the mixer for 4 min. The slump was measured to be 200 mm at that time, then the slurry was loaded into a cylindrical die of Φ50 × 100 mm^2^, vibrated on a shaking table for 30 s and placed in a standard constant temperature and humidity curing box (SHBY-90B, Cangzhou, Hebei, China) for one day. The specimen was cured for a certain period of time at a temperature of 20 ± 2 °C and relative humidity of 95 ± 2% before use.

#### 2.2.2. Combined Chloride and Wet–Dry Cycles Erosion Test

According to the Standard for Test Method for the Long-term Performance and Durability of Ordinary Concrete (GB/T 50082-2009), oven drying and room temperature soaking were used to simulate alternating dry and wet conditions. After curing for 26 days, the specimen was removed, wiped dry and dried for 48 h in an oven at 80 ± 5 °C. It was removed from the oven and cooled to room temperature before it was placed in a PVC box. The specimen was soaked in 0 (clean water), 5% and 10% chloride solutions for 12 h. The solution was removed, and the specimen was placed in an oven. The solution was baked at 80 ± 5 °C for 12 h.

#### 2.2.3. Uniaxial Compressive Strength Test

According to the Standard for Test Methods for the Physical and Mechanical Properties of Concrete (GB/T 50081-2019), the specimens were tested for uniaxial compressive strength by a microcomputer-controlled electronic universal testing machine (WDW-10E, Jinan, Shandong, China) after the cycling period reached 0, 2, 4, 6, 8, 10 and 12 cycles. The loading rate was 1 mm/min. Five specimens were measured in each group of tests, and the average of 5 test values was taken as the final result (accurate to 0.001 MPa).

#### 2.2.4. Toxic Substance Leaching Test

A toxic substance leaching test was carried out using the Toxic Leaching Method of Solid Waste Leaching-Horizontal Oscillation Method (HJ 557-2010). First, after 0, 2, 4, 6, 8, 10 and 12 cycles of leaching, specimens were dried in an oven, broken into pieces and sieved through a 3 mm sieve. A 10 g sample was placed in a conical flask, deionized water was added to yield a liquid–solid ratio of 10:1 (L/g) and the flask was sealed with plastic wrap and placed in a horizontal oscillating device (BSD-TX370, Shanghai, China). The oscillation frequency was set to 110 r/min, the amplitude was set to 40 mm and the temperature was set to 25 ± 0.5 °C. After 8 h of oscillation, it was left undisturbed for 16 h. Then, 50 mL of supernatant was extracted with a syringe and placed in a beaker, followed by acidification with 1% concentrated HNO_3_ solution (67% mass fraction). The leached ion concentration was measured in a colorimetric tube using an atomic absorption spectrophotometer (Hitachi Z-2000, Tokyo, Japan).

#### 2.2.5. Microscopic Analysis

After the cycling period, the filling paste samples were broken into small pieces and immersed in anhydrous ethanol in a 50 mL PVC bottle. After the hydration reaction was complete, the samples were dried at 80 ± 5 °C for 12 h. Scanning electron microscopy (JSM-7500F, Tokyo, Japan) was used to analyze the microstructure and morphology of the hydration products. X-ray diffraction (XRD-6100, Kyoto, Japan) was conducted at a rate of 4.5‰ to analyze the phase composition of the samples.

## 3. Results and Discussion

### 3.1. Synergies among Solid Waste

**Figure 3:**
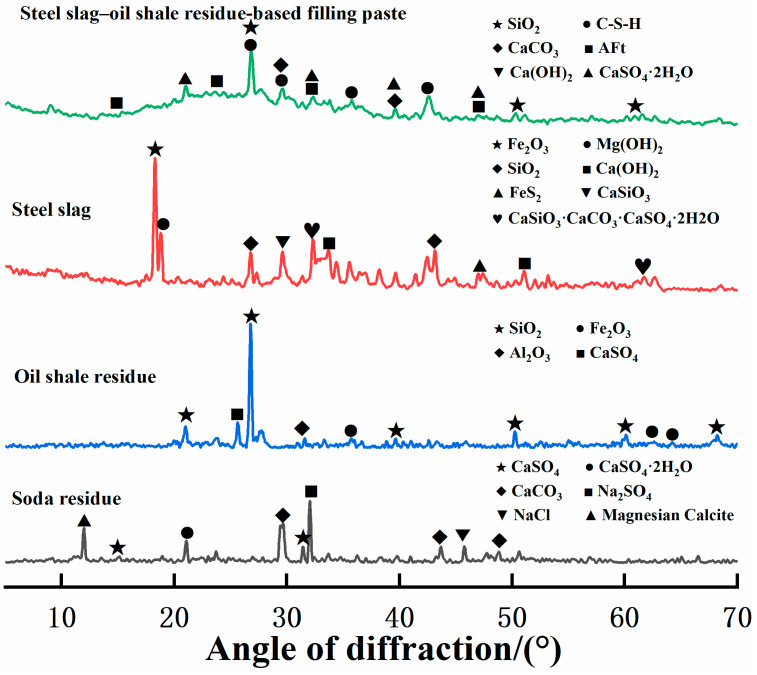
XRD comparison of raw materials and steel slag–oil shale residue-based filling paste.

**Figure 4:**
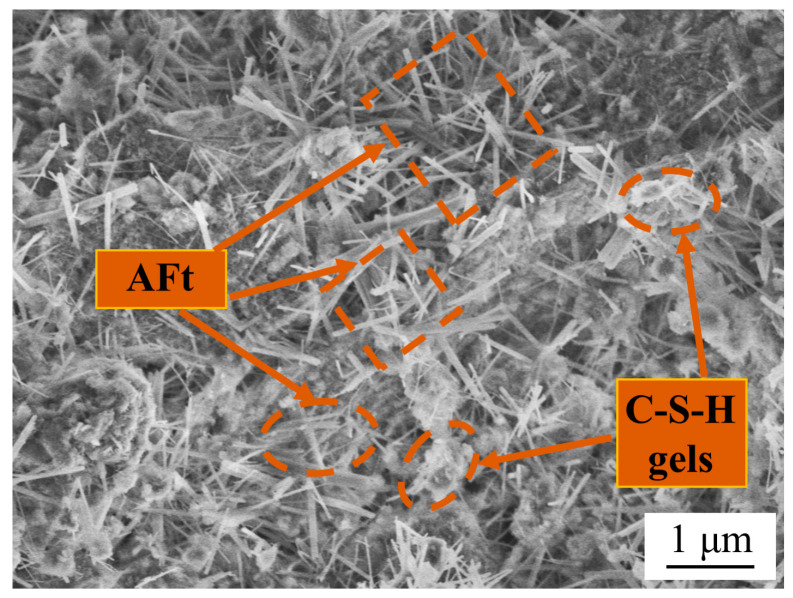
SEM of steel slag–oil shale residue-based filling paste.

Since the steel slag–oil shale residue-based filling paste is only composed of steel slag, soda residue, oil shale residue and water and does not contain cement or other cementing agents or alkali activators, a cemented state is formed, and strength is generated by the interaction between the solids and waste. To explore the mechanism of strength formation, XRD tests were conducted on the raw materials, and SEM and XRD tests were conducted on specimens after 28 days of standard curing. The phase composition, structure and morphology of the hydration products are shown in Figure 3 and Figure 4.

**Figure 5:**
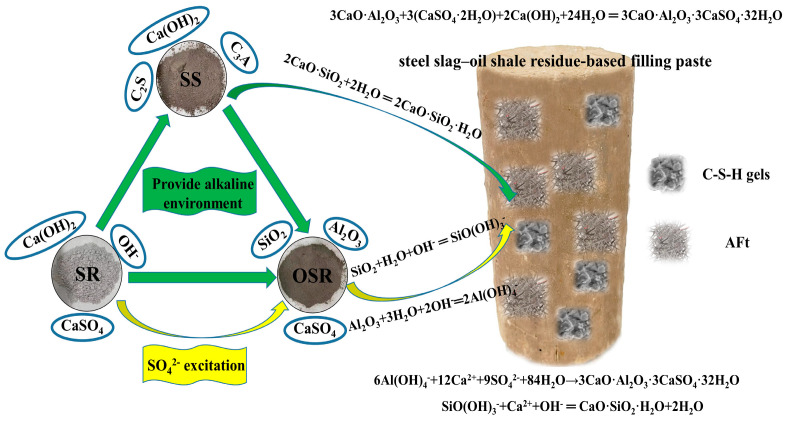
Strength formation mechanism of steel slag–oil shale residue-based filling paste.

According to Figure 3 and the chemical composition of the aforementioned raw materials, steel slag provides CaO, C_2_S and C_3_A for the hydration reaction. Oil shale residue provides CaSO_4_ and plenty of Al_2_O_3_ and SiO_2_; soda residue provides CaO, CaSO_4_ and OH^−^. C_3_A reacts with CaSO_4_ and Ca(OH)_2_ in water to form ettringite (AFt). At the same time, due to the strong oxidation ability of OH^−^, the active Al_2_O_3_ and active SiO_2_ in the oil shale residue undergo a complexation reaction in an alkaline environment, SiO(OH)^−^ and Al(OH)_4_^−^ are generated and SiO(OH)^−^ and Al(OH)_4_^−^ hydrate with CaSO_4_ and Ca(OH)_2_, respectively, promoting the formation of AFt and C-S-H gels in the material. Needle-rod-shaped AFt and flock-like C-S-H gels are shown in Figure 4. The formation of AFt and C-S-H gels increased cementation strength and filled internal pores. Therefore, the hydration reaction similar to cement in Figure 5 is generated through the synergistic and complementary action of the components in the three solid waste materials.

### 3.2. Analysis of Compressive Strength

**Figure 6:**
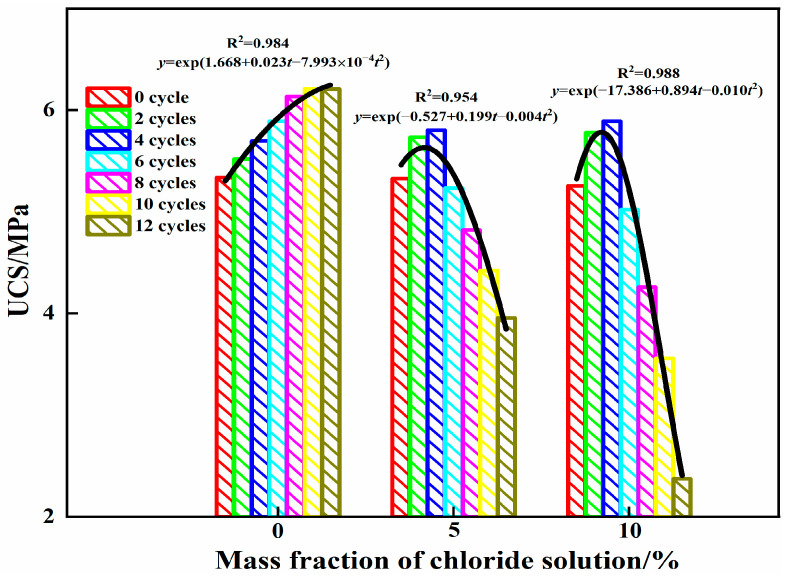
Changes in compressive strength and fitting curves of steel slag–oil shale residue-based filling paste.

Figure 6 shows the relationship between the filling paste strength, the mass fraction of the chloride solution and the cycling period under combined erosion driven by chloride and wet–dry cycles. As shown in Figure 6, with the increasing number of cycles, the compressive strength of specimens in the 0% chloride solution first increased and then tended to remain stable, while the strength of the specimens in the 5% and 10% chloride solutions first increased, then decreased and reached a maximum in the fourth cycle. Under the same cycle conditions, the compressive strength of the specimens increased with increasing mass fraction of the chloride solution in the first four cycles and decreased with increasing mass fraction of the chloride solution after six cycles.

In the first four cycles, the compressive strength of the specimen increased gradually with increasing cycling in the 0% chloride solution. The main reason is that the AFt and C-S-H gels generated during the secondary hydration reaction that occurred during the dry–wet cycles filled internal pores and improved compactness, which was manifested as an increase in compressive strength on a macroscopic level. Compared with the 0% chloride solution, the compressive strength of the specimens increased faster in 5% and 10% chloride solutions. The reason for this phenomenon may be that Cl^−^ penetrated into the specimen and generated Friedel’s salt, which filled the internal pores. As more hydration products were generated in the 10% chloride solution than in the 5% chloride solution, the compressive strength increased with increasing mass fraction of chloride in the solution.

After six cycles, the variation in the compressive strength of the specimens in the 0% chloride solution was significantly different from those in the 5% and 10% chloride solutions. The compressive strength of the specimens increased slowly and tended to remain stable in the 0% chloride solution because the hydration reaction nearly ceased to occur. The compressive strength of the specimens decreased from 5.231 and 5.021 MPa to 3.954 and 2.371 MPa when the mass fraction of chloride salt in the solution was 5% and 10%, respectively. This is because with an increase in the number of dry–wet cycles and continuous erosion by Cl^−^, more hydration products accumulated inside the specimen, resulting in surface spalling and the formation of local cracks, reducing the compressive strength of the specimen. The erosion of specimens in the 10% chloride solution was more severe than that in the 5% chloride solution, and strength decreased faster. The fitted relationship between the compressive strength and cycle number is shown in Figure 6. In the figure, y is the uniaxial compressive strength, and t is the cyclic period. Within 12 cycles, the compressive strength of the specimens followed an exponential function as the mass fraction of chloride in the solution changed. The compressive strength of the steel slag–oil shale residue-based filling paste was related to the chloride mass fraction and cycling period of the chloride solution, and its strength can be expressed as Equation (1):

(1) $$\sigma_{t}=e^{a+bt+ct^{2}}$$

where *σ*_t_ is the uniaxial compressive strength of the filling paste, *t* is the cycling period and *a*, *b* and *c* are the fitting coefficients related to the mass fraction of chloride salt in the solution and the cycling period.

### 3.3. Analysis of Leaching Characteristics

**Figure 7:**
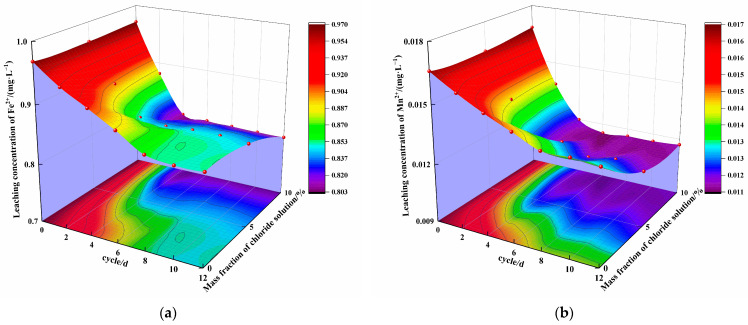

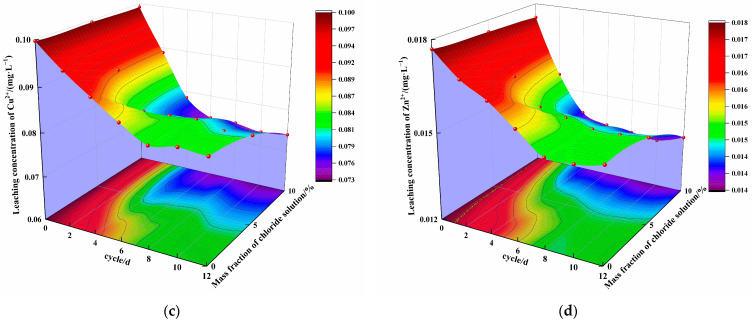
Leached concentrations of (**a**) Fe^2+^, (**b**) Mn^2+^, (**c**) Cu^2+^ and (**d**) Zn^2+^ inside the steel slag–oil shale residue-based filling paste.

Figure 7 shows the relationship between the concentrations of Fe^2+^, Mn^2+^, Cu^2+^ and Zn^2+^ in the leaching solution of the steel slag–oil shale residue-based filling paste, the mass fraction of chloride salt in the solution and the cycling period under combined erosion from chloride and wet–dry cycles.

The leached concentrations of Fe^2+^, Mn^2+^, Cu^2+^ and Zn^2+^ in the 0% chloride solution decreased by 12.03%, 16.87%, 16.75% and 14.12% compared with the initial concentrations, respectively. The leached concentrations of Fe^2+^, Mn^2+^, Cu^2+^ and Zn^2+^ in the 5% chloride solution decreased by 12.78%, 27.88%, 19.10% and 14.77% compared with the initial concentrations, respectively. The leached concentrations of Fe^2+^, Mn^2+^, Cu^2+^ and Zn^2+^ in the 10% chloride solution decreased by 17.19%, 30.36%, 25.40% and 20.00%, respectively. The higher the mass fraction of chloride in the solution, the more hydration products accumulated and the lower the ion leaching concentration.

As shown in Figure 7, with increasing cycling with the 0% chloride solution, the concentration of all ions in the leaching solution first slowly decreased and then tended to remain stable, while the concentration of all ions in the leaching solution of specimens with 5% and 10% chloride solutions first rapidly decreased and then tended to remain stable. Under the same cycling conditions, the concentration of ions in the leaching solution decreased with increasing chloride content. This is because secondary hydration reactions occurred inside the specimen in the 0% chloride solution in the early stage, resulting in C-S-H gels with extremely large specific surface area and surface energy, which could adsorb metal ions and slowly reduce the leached ion concentration. In the later stage, the hydration reaction nearly ceased to occur, and the leached ion concentration tended to remain stable. The concentration of ions in the leaching solution of specimens with mass fractions of 5% and 10% chloride salt decreased rapidly first and then tended to remain stable. This occurred because in the early stage of cycling, under the action of Cl^−^, a hydration reaction occurred in the specimen to generate Friedel’s salt, which can solidify heavy metal ions and reduce the leached concentration of ions rapidly. With an increase in the number of cycles, a large number of hydration products accumulated, and the ion concentration in the leaching solution tended to remain stable. This is different from the trend observed in a conventional test for solidified contaminated soil [18]. Zha et al. [19] believed that when the concentration of pollutant ions is high, heavy metal ions can not only hinder the formation of hydration products of materials but also may exceed the adsorption capacity of hydration colloids. When the concentration of pollutant ions is low, most metal ions can be immobilized in the specimen, and the ion concentration in the leaching solution tends to remain stable, although some metal ions are dissolved from localized cracks in the specimen. As the content of pollutants in the steel slag–oil shale residue-based filling paste developed in this study is low, the leached concentration of pollutants in the later stage did not increase but tended to remain stable.

### 3.4. Microscopic Characterization

#### 3.4.1. Phase Composition of Steel Slag–Oil Shale Residue-Based Filling Paste

**Figure 8:**
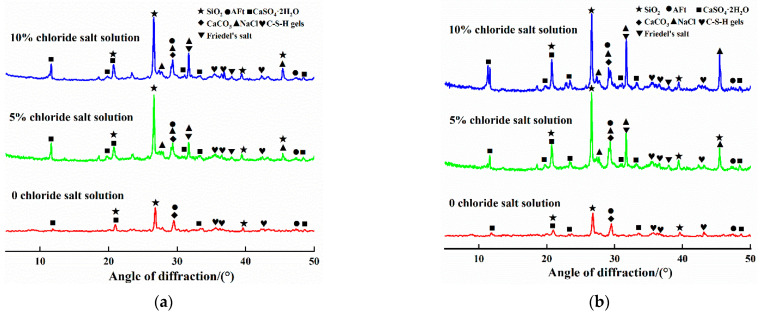
XRD pattern of specimen eroded by the combined action of chloride and dry–wet cycling. (**a**) 2 cycles and (**b**) 12 cycles.

To investigate the phase composition of the internal erosion products of the steel slag–oil shale residue-based filling paste, XRD was used to analyze select samples. Figure 8 shows the XRD patterns of a specimen after 2 and 12 wet–dry cycles in the 0, 5% and 10% chloride solutions.

According to Figure 8a, after two cycles, the main phases of the specimen in the solution with 0% chloride were quartz (SiO_2_), ettringite (AFt), calcite (CaCO_3_) and gypsum (CaSO_4_·2H_2_O). In contrast, with an increase in the mass fraction of chloride in the solution, NaCl diffraction peaks appeared in the XRD patterns, indicating that Na^+^ and Cl^−^ penetrated the specimen when the specimen was immersed in the chloride salt solution, and some white crystals precipitated on the surface of the specimen when the interior was saturated. At the same time, Friedel’s salt was found in the diffraction peaks, and Friedel’s salt could fill the internal pores in the specimen in the early stages of cycling; therefore, at the macroscopic level, an increase in strength was observed. Compared with the results in Figure 8a, when the solution had been cycled 12 times (Figure 8b), there was no change in the main phase types in the XRD pattern. Friedel’s salt and sodium chloride diffraction peaks had stronger diffraction peaks, indicating that there were more Na^+^ and Cl^−^ ions in the specimen, which led to an enhancement in the sodium chloride diffraction peaks and the precipitation of more sodium chloride crystals on the surface, and a large amount of Cl^−^ participated in the hydration reaction. Friedel’s salt production increased, local cracks appeared in the specimen and strength decreased.

#### 3.4.2. Microstructure of Steel Slag–Oil Shale Residue-Based Filling Paste

**Figure 9:**
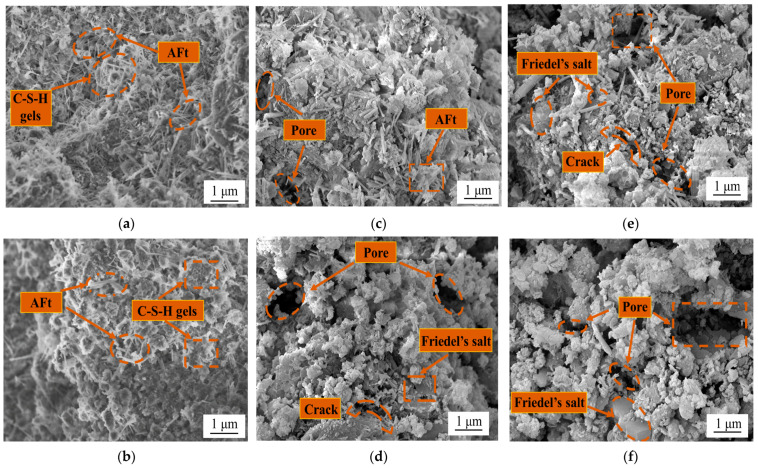
SEM images of specimens in (**a**) 2 dry-wet cycles with 0 chloride content, (**b**) 12 dry-wet cycles with 0 chloride content, (**c**) 2 dry-wet cycles with 5% chloride content, (**d**) 12 dry-wet cycles with 5% chloride content, (**e**) 2 dry-wet cycles with 10% chloride content and (**f**) 12 dry-wet cycles with 10% chloride content.

Figure 9 shows the SEM image of the specimen after it underwent 2 and 12 wet–dry cycles in 0%, 5% and 10% chloride solution. The specimens in the 0% chloride solution are shown in Figure 9a,b. There were micropores of other sizes in the specimen, and different amounts of needle-rod-shaped AFt and flock-like C-S-H gels existed in different cycles, filling in the internal microfractures of the specimen. With an increase in the number of cycles, the number of pores decreased, and the internal structure became tighter. Figure 9c,d are SEM images of the specimen after 2 and 12 wet–dry cycles in the 5% chloride solution. With an increase in Cl^−^ in the solution, obvious holes, microseams and needle-rod-shaped AFt appeared in the interior in the early stage of erosion, while it was observed that the holes and microseams further expanded and AFt decreased significantly in the later stage, and a small amount of flake-like Friedel’s salt was formed. The microstructure of the specimen in the 10% chloride solution is shown in Figure 9e,f. The erosion became more obvious with increasing mass fraction of chloride in the solution. Flaky Friedel’s salt and pores were observed in the early stage of erosion. In the later period, the holes were further enlarged, and Friedel’s salt was more abundant.

## 4. Analysis and Discussion

### 4.1. Effect of Chloride and Wet–Dry Cycling-Driven Erosion on the Strength of Steel Slag–Oil Shale Residue-Based Filling Paste

Based on the above SEM and XRD analysis of the steel slag–oil shale residue-based filling paste, it can be found that because Cl^−^ has strong permeability [20], it first combines with small, residual amounts of Ca(OH)_2_ in the interior of the specimens and then reacts with C_3_A and other hydration products to produce Friedel’s salt. In the early stage of erosion, Friedel’s salt fills internal crevices, leading to an increase in strength. During prolonged cycling under erosive conditions, continuous immersion in the chloride salt leads to the formation of a large amount of Friedel’s salt, leading to the formation of local cracks and resulting in a decrease in strength at the macroscopic level.

The steel slag–oil shale residue-based filling pastes are similar to traditional cement-based filling pastes in that the erosive action of chloride and wet–dry cycles is related to Ca(OH)_2_, but the difference is that the source and content of Ca(OH)_2_ are different. The Ca(OH)_2_ in the steel slag–oil shale residue-based filling paste is provided by steel slag and soda residue. The alkaline environment created by steel slag and soda residue enables SiO_2_ and Al_2_O_3_ in oil shale residue to carry out continuous hydration reactions, and a large amount of Ca(OH)_2_ is consumed in the hydration stage. When Cl^−^ enters the specimen, the hydration reaction consumes a large amount of Ca(OH)_2_, making the cemented paste backfill consisting of total solid wastes more resistant to chloride erosion. See Equations (2) and (3) for the chemical equations for each reaction.

(2) $${\mathrm{Ca}(\mathrm{OH})}_{2}+2\mathrm{NaCl}\begin{array}{c} \underline{\underline{\;\;\;\;\;}} \end{array}{\mathrm{CaCl}}_{2}{+2\mathrm{Na}}^{+}{+2\mathrm{OH}}^{-}$$

(3) $$3\mathrm{CaO}\cdot {\mathrm{Al}}_{2}O_{3}{+\mathrm{CaCl}}_{2}{+10H}_{2}O\begin{array}{c} \underline{\underline{\;\;\;\;\;}} \end{array}3\mathrm{CaO}\cdot {\mathrm{Al}}_{2}O_{3}\cdot {\mathrm{CaCl}}_{2}\cdot {10H}_{2}O$$

### 4.2. Effect of Erosion under the Combined Action of Chloride and Wet–Dry Cycling on the Leaching Characteristics of Steel Slag–Oil Shale Residue-Based Filling Paste

The immobilization of metal ions by hydration products generated in steel slag–oil shale residue-based filling paste mainly involves physical and chemical mechanisms. Physical action, namely, physical adsorption, is realized by intermolecular van der Waals forces. Chemical processes include surface complexation, ion exchange and precipitation. The immobilization of heavy metal ions (Mn^2+^, Zn^2+^, Fe^2+^ and Cu^2+^) in steel slag–oil shale residue-based filling paste is different under combined erosion from chloride and wet–dry cycling. Surface complexation, ion exchange and precipitation of metal ions with AFt, C-S-H gels, Friedel’s salt and other hydration products occurred.

**Figure 10:**
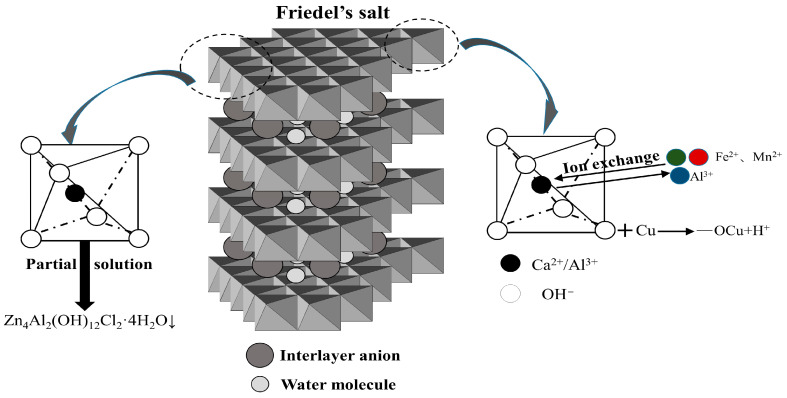
Solidification effect of Friedel’s salt on metal ions.

The Friedel’s salt plate structure is composed of metal ions and six hydroxyl groups forming octahedral coedges [21]. Mn^2+^ and Fe^2+^ are mainly involved in ion exchange, which can replace octahedrally coordinated Al in the crystal structure of Friedel’s salt [22,23]. Zn^2+^ and Cu^2+^ undergo surface complexation and surface precipitation reactions [22,24,25]. Cu^2+^ mainly combines with surface OH, and due to the partial dissolution of Friedel’s salt at room temperature [26], Zn^2+^ mainly combines with Al(OH)_4_^−^, OH− and Cl^−^ to precipitate. The diagram of the overall reactions is shown in Figure 10.

In addition, in AFt, Zn^2+^, Cu^2+^ and Mn^2+^ can be fixed internally in a columnar lattice [27], and Zn^2+^ and Cu^2+^ can replace Ca^2+^ in the C-S-H gels to produce ZnH_2_SiO_4_ and CuH_2_SiO_4_ [28].

## 5. Conclusions

The effect of paste filling on the environment should be considered, and this can directly determine a paste’s application potential and safety. In this paper, steel slag–oil shale residue-based filling paste was taken as the research object, and variations in the compressive strength and leaching characteristics of the steel slag–oil shale residue-based filling paste in a complex environment was studied through combined chloride and wet–dry cycling erosion experiments, and changes in microstructure were analyzed. The conclusions are as follows:

(1) 1. The influence of the mass fraction of chloride in the solution and the number of cycles on the uniaxial compressive strength of steel slag–oil shale residue-based filling paste is obvious. Within 12 cycles, AFt and C-S-H gels increased in the aqueous solution with 0% chloride, resulting in gradual internal compactness and increased compressive strength. When the specimen was immersed in 5% and 10% chloride salt solutions, Friedel’s salt was generated due to erosion by Cl^−^ in the early stages of cycling, which filled internal pores, increased compressive strength and resulted in peak strength values. With an increase in the number of cycles, large amounts of hydration products accumulated in the specimen in the later stage, resulting in the formation of local cracks and decreasing compressive strength. The compressive strength of the specimen had an exponential relationship with the cycling period.
2. Over 12 cycles, the concentration of ions in the specimen leaching solution with 0% chloride first slowly decreased and then tended to remain stable, while the concentration of ions in the specimen leaching solution with mass fractions of 5% and 10% chloride salt first rapidly decreased and then tended to remain stable. Under the same cycling conditions, the concentration of ions in the leaching solution decreased with increasing mass fraction of chloride in the solution. The leached concentration of all ions decreased compared with the initial concentration. The number of cycles and the mass fraction of chloride affect the type and quantity of erosion products, playing a role in curing heavy metal ions.
3. Ca(OH)_2_ in the steel slag–oil shale residue-based filling paste is provided by steel slag and soda residue. The alkaline environment created by steel slag and soda residue enables SiO_2_ and Al_2_O_3_ in the oil shale residue to carry out continuous hydration reactions, and a large amount of Ca(OH)_2_ is consumed in the hydration stage. However, with an increase in the number of dry–wet cycles and due to continuous immersion in chloride salts, the hydration reactions of the traditional cement-based filling materials progress adequately, and an increasing amount of Ca(OH)_2_ is generated. This indicates that the steel slag–oil shale residue-based filling paste has stronger chloride resistance than traditional cement-based filling pastes.

## Figures and Tables

**Table 1:** Main mineral components (mass fraction) of steel slag, oil shale residue and soda residue.

Materials	Al_2_O_3_	CaO	SiO_2_	Fe_2_O_3_	MgO	SO_3_	Na_2_O	TiO_2_	K_2_O	MnO	P_2_O_5_	Cl
Steel slag	6.15	40.75	12.39	14.17	21.21	2.79	0.03	0.81	0.15	1.23	—	—
Oil shale residue	14.71	7.15	62.16	8.32	2.38	0.82	0.04	0.87	3.07	0.24	—	—
Soda residue	4.31	66.12	9.68	0.29	0.31	11.21	1.99	0.22	1.32	—	0.18	2.62

**Table 2:** Leached ion concentrations of steel slag, oil shale residue and soda residue(mg/L).

Ions	Fe^2+^	Mn^2+^	Cu^2+^	Zn^2+^
Steel slag	1.503	0.123	0.231	0.154
Oil shale residue	1.111	0.087	0.101	0.077
Soda residue	0.455	0.048	0.012	0.012

## Data Availability

Data can be obtained from corresponding authors upon reasonable request.

## References

[1] Cao S., Song W., Xue G., Ma R., Zhu P. (2016). Mechanical characteristics variation of stratified cemented tailing backfilling and its failure modes. J. China Univ. Min. Technol..

[2] Liu L., Wang S., Zhu M., Zhang B., Hou D., Xun C., Zhao Y., Zhang X., Wang X., Wang M. (2022). CO_2_ storage-cavern construction and storage method based on functional backfill. J. China Coal Soc..

[3] Liu S., Wang F., Li G., Liu G., Wang J., Qi Z. (2021). Optimization of mixture ratio and microstructure influence mechanism of composite filling slurry based on response surface method. Acta Mater. Compos. Sin..

[4] Lan W., Wu A., Wang Y. (2019). Formulation optimization and formation mechanism of condensate expansion and filling composites. Acta Mater. Compos. Sin..

[5] Zhao K., He Z., Ning F., Zhou Y., Yan Y., Wang Y., Wang J. (2021). Acoustic emission characteristics of cementitious material with different cement-tailing ratio. J. Chin. Ceram. Soc..

[6] Ruan Z., Wu A., Wang Y., Wang S., Wang J. (2022). Multiple response optimization of key performance indicators of cemented paste backfill of total solid waste. Chin. J. Eng..

[7] Li B., Yin H., Mao X., Li Y., Zhang L., Liu R., Qiu P. (2016). Macroscopic and microscopic fracture features of concrete used in coal mine under chlorine salt erosion. Int. J. Min. Sci. Technol..

[8] Sun Q., Li X., Wei X., Mu Q. (2015). Experimental study of the influence of chloride corrosion on creep properties of filling paste. J. Exp. Mech..

[9] Ma D., Kong S., Li Z., Zhang Q., Wang Z., Zhou Z. (2021). Effect of wetting-drying cycle on hydraulic and mechanical properties of cemented paste backfill of the recycled solid wastes. Chemosphere.

[10] Du Z., Chen S., Yin D., Yao D., Zhang Z. (2021). Experimental study of stability of paste backfill under chloride erosion environment. J. China Univ. Min. Technol..

[11] Zhou X., Liu C., Feng B., Guo B., Lu Y., Zhang L. (2019). Effects of dry-wet circulation on cement-based composite filling materials. Chin. J. Eng..

[12] Wang S., Wang F., Yin D., Jiang T., Zhang Z. (2021). Experimental Study on Mechanical Properties of Paste Backfill with Flue-Gas Desulphurisation Gypsum under Combined Action of Dry–Wet Cycles and Chloride Erosion. Minerals.

[13] Liu H., Zhang J., Zhou N., Shen L., Zhang L., Zhu C., Wang L. (2021). Study of the leaching and solidification mechanism of heavy metals from gangue-based cemented paste backfilling materials. J. China Univ. Min. Technol..

[14] Chen Q., Zhang Q., Qi C., Zhang Q., Feng Y., Wang D., Tao Y., Lu R. (2021). Temperature-depending characteristics of strength and leaching toxicity of phosphogympsum-based cemented paste backfill. Chin. J. Nonferrous Met..

[15] Mahedi M., Cetin B. (2021). Leaching of elements from cement activated fly ash and slag amended soils. Chemosphere.

[16] Yang H., Ni W., Zhang S., Ma X. (2018). Mechanisms of solidification of cadmium in municipal solid waste incineration fly ash usinga slag cemented backfill agent. Chin. J. Eng..

[17] Li K., Li X., Du C., Xue H., Sun Q., Liu L. (2022). Experimental study on microstructure and erosion mechanisms of solid waste cemented paste backfill under the combined action of dry–wet cycles and sulphate erosion. Materials.

[18] Zhang H., Yang Y., Yi Y. (2017). Effect of sulfate erosion on strength and leaching characteristic of stabilized heavy metal contaminated red clay. Trans. Nonferrous Met. Soc. China.

[19] Zha F., Liu J., Xu L., Cui K. (2013). Cyclic wetting and drying tests on heavy metal contaminated soils solidified/stabilized by cement. Chin. J. Geotech. Eng..

[20] Malliou O., Katsioti M., Georgiadis A., Katsiri A. (2007). Properties of stabilized/solidified admixtures of cement and sewage sludge. Cem. Concr. Compos..

[21] Zhang J. (2013). Study on Synthesis, Characterization of Ca-Based Functional Materials and Their Adsorption of Heavy Metal Ions. Master’s Thesis.

[22] Xia Z., Wang M., Wang F., Shang D. (2017). Formation process of interstitials containing manganese and its solidification. J. Chin. Ceram. Soc..

[23] Wang X., Wang Q., Zhang B., Ni W., Jin R., Zhao K. (2018). Hydration mechanism of using steel slag as binder for backfill materials in potash mines. Chin. J. Eng..

[24] Liu Q., Li Y., Zhang J., Chi Y., Ruan X., Liu J., Qian G. (2011). Effective removal of zinc from aqueous solution by hydrocalumite. Chem. Eng. J..

[25] Park M., Choi C., Seo Y., Yeo S., Choi J., Komarneni S., Lee J. (2007). Reactions of Cu^2+^ and Pb^2+^ with Mg/Al layered double hydroxide. Appl. Clay Sci..

[26] Bothe J., Brown P. (2004). PhreeqC modeling of Friedel’s salt equilibria at 23 ± 1 °C. Cem. Concr. Res..

[27] Qian G., Cao Y., Chui P., Tay J. (2006). Utilization of MSWI fly ash for stabilization/solidification of industrial waste sludge. J. Hazard. Mater..

[28] Mijno V., Catalan L., Martin F., Bollinger J. (2004). Compositional changes in cement-stabilized waste during leach tests-comparison of SEM/EDX data with predictions from geochemical speciation modeling. J. Colloid Interface Sci..

